# Beyond the Biopsy: Imaging Frontiers in Cardiac Sarcoidosis

**DOI:** 10.1155/crp/3738354

**Published:** 2026-04-17

**Authors:** Ibrahim Antoun, Jalal Assadi, Mustafa Zakkar, G. Andre Ng, Sanjay S. Bhandari

**Affiliations:** ^1^ Department of Cardiology, University Hospitals of Leicester NHS Trust, Leicester, UK, nhs.uk; ^2^ Division of Cardiovascular Sciences, College of Life Sciences, University of Leicester, Leicester, UK, le.ac.uk; ^3^ Department of Radiology, University Hospitals of Leicester NHS Trust, Leicester, UK, nhs.uk; ^4^ Leicester British Heart Foundation Centre of Research Excellence, Leicester, UK

**Keywords:** cardiac magnetic resonance, cardiac sarcoidosis, FDG-PET CT, multimodality imaging, myocardial inflammation

## Abstract

Cardiac sarcoidosis (CS) represents an inflammatory infiltrative disease, which creates difficulties during diagnosis and prognosis. The identification and management of CS heavily depend on advanced cardiac imaging techniques. The article examines multimodality imaging in CS by evaluating cardiac magnetic resonance (CMR) imaging, positron emission tomography (PET) with computed tomography (CT), single‐photon emission computed tomography (SPECT), echocardiography, CT and hybrid imaging approaches. This review examines each imaging modality with respect to its fundamental principles, diagnostic capabilities, benefits and drawbacks and references key research from the past decade. CMR stands as the primary diagnostic method for detecting myocardial scar and fibrosis in CS patients, providing essential prognostic information. The PET/CT system with 18‐fluorodeoxyglucose (FDG) provides an effective method for detecting active inflammation and tracking treatment response. Echocardiography is a readily accessible screening tool that reveals the structural and functional effects of CS, while strain imaging techniques enable the detection of early disease involvement. The use of delayed‐enhanced cardiac CT provides an alternative method for detecting myocardial scar. It helps identify patients who are ineligible for CMR and excludes coronary artery disease. Diagnostic confidence and disease activity assessment improve with hybrid imaging, particularly when PET/MR is used. The Discussion section integrates these findings with an evaluation of emerging quantitative imaging markers and novel tracers that show promise for enhancing CS evaluation. The implementation of multimodality imaging techniques has transformed the management of CS by enabling earlier diagnosis, risk assessment and treatment guidance. A personalised imaging approach that combines multiple diagnostic methods yields the best results for diagnosing CS and enhances patient‐care outcomes.

## 1. Introduction

Sarcoidosis is a granulomatous disease that affects the heart muscle, resulting in cardiac sarcoidosis (CS). The manifestation of clinically evident CS occurs in less than 5% of sarcoidosis patients although autopsy findings indicate cardiac involvement affects between 20% and 25% of the patients [1]. The early detection of CS remains vital because myocardial involvement can manifest as high‐grade atrioventricular block, myopericarditis, progressive heart failure or life‐threatening ventricular arrhythmias and sudden cardiac death [2, 3]. The noncaseating granuloma pattern is challenging to diagnose, as endomyocardial biopsy has low sensitivity (< 25%) and yields patchy results [4]. Recent developments in cardiac imaging methods have substantially enhanced the detection and management capabilities for patients with CS. The current consensus criteria, established by the Heart Rhythm Society (HRS) in 2014 [5] and the Japanese Circulation Society in 2017 [6], utilise late gadolinium enhancement (LGE) on Cardiac magnetic resonance (CMR) and focal fluorodeoxyglucose‐18F (FDG) uptake on positron emission tomography (PET) as their primary diagnostic indicators [7, 8]. Detection of CS now relies primarily on noninvasive imaging methods, which are the standard diagnostic tools when tissue samples cannot be obtained.

Transthoracic echocardiography (TTE) is typically the first‐line cardiac imaging modality because of its wide availability and rapid bedside assessment of cardiac structure and function. Although TTE cannot directly visualise myocardial granulomas, fibrosis or active inflammation, echocardiographic abnormalities are reported in approximately 14%–46% of the patients with systemic sarcoidosis, even in the absence of cardiac symptoms or electrocardiographic abnormalities [9]. Reported findings include regional wall motion abnormalities in noncoronary distributions, basal septal thinning or aneurysm formation, left or right ventricular (RV) systolic and diastolic dysfunction, ventricular dilatation and pericardial effusion. Furthermore, advanced imaging modalities play a central role in diagnosis and risk stratification. CMR enables visualisation of myocardial fibrosis, scar formation and oedema, providing important prognostic information. The diagnostic assessment and monitoring of disease activity rely heavily on PET with FDG, which detects active myocardial inflammation via metabolic activity. Gallium scintigraphy and single‐photon emission computed tomography (SPECT) perfusion imaging retain historical significance but have largely been supplanted by PET owing to superior sensitivity and spatial resolution. Delayed contrast‐enhanced cardiac computed tomography (CT) allows detection of myocardial scar in patients with implanted devices or contraindications to CMR. Hybrid imaging with PET and magnetic resonance (PET/MR) enables simultaneous assessment of fibrosis and inflammation in a single examination, further improving diagnostic confidence and assessment of disease activity.

This article examines each imaging approach used for CS through a detailed review of its fundamental concepts, diagnostic applications, benefits and drawbacks and major findings from the past decade. The following discussion explains how these imaging methods work together as part of a combined diagnostic approach and introduces new developments which will shape future CS imaging practices. The authors aim to guide clinical professionals and imaging specialists toward optimal cardiac imaging approaches for sarcoidosis, thereby facilitating faster diagnosis and improved disease management.

## 2. Echocardiography

TTE offers multiple benefits: it provides noninvasive results that are readily accessible at the bedside, making it suitable for both initial assessments and follow‐up care. It serves as the initial tool for detecting cardiac involvement in patients with extracardiac sarcoidosis, according to the HRS. A normal echocardiogram cannot rule out CS since it lacks sufficient sensitivity to detect all CS cases [5]. TTE is the primary imaging modality for patients suspected of CS due to its availability, real‐time assessment of cardiac structure and function and portability [5]. TTE lacks tissue‐specific sensitivity compared with CMR and PET, yet it detects numerous macroscopic changes characteristic of sarcoid involvement. TTE can be normal in CS; however, it reveals several findings related to CS, including wall motion abnormalities in noncoronary areas, septal or ventricular wall thinning or aneurysms, systolic and diastolic dysfunction, RV dysfunction and intracardiac aneurysms [10–12]. The presence of discrete basal septal thinning or an aneurysm is a classic sign of CS involvement, as is dilatation in advanced stages of the disease [13, 14]. Sarcoidosis may cause pericardial effusion in the affected heart by leading to pericardial involvement and inflammation. Rarely, CS may present with mass‐like myocardial thickening or focal infiltrative lesions, usually reflecting advanced granulomatous involvement; however, such appearances are uncommon, nonspecific and are not a typical or reliable echocardiographic feature [15]. Studies have demonstrated that TTE has limited sensitivity for diagnosing CS when used in isolation. For example, Kouranos et al. reported that the addition of echocardiography to clinical history and electrocardiography identified only approximately 27% of the patients who met diagnostic criteria for CS [16], highlighting its limited diagnostic yield as a standalone test. Nevertheless, echocardiography remains an essential first‐line and follow‐up imaging modality for functional assessment and exclusion of alternative cardiac pathology.

The recent development of speckle‐tracking echocardiography (STE) has enabled the detection of subtle myocardial dysfunction in patients with CS. Through STE, healthcare providers can measure deformation (strain) in various directions throughout the heart [17]. Assessment of left ventricular global longitudinal strain (GLS) is a sensitive measure of cardiac contractility, which typically declines before ejection fraction (EF). The presence of decreased GLS values in sarcoidosis patients without EF reduction may indicate initial involvement of the disease. GLS measurements in sarcoidosis patients with no known CS and normal EF were significantly lower than those in healthy controls (−17.2% vs. −21.3%; *p* < 0.001) [18]. The development of adverse outcomes or the manifestation of CS was more common among patients with higher strain values. Di Stefano et al. found equivalent outcomes in their research when studying sarcoid patients with normal EF, as those with CS had lower absolute left ventricular global longitudinal strain (LV GLS) (−16%) compared with those without CS (−18%), as well as decreased RV free‐wall strain [17]. CS causes strain reductions primarily in the basal septum or lateral wall, yet global strain results are most commonly reported [19]. Healthcare professionals can calculate strain‐based dispersion indices or regional strain abnormalities. The “strain mapping” method enables investigators to identify specific basal, lateral and septal deficiencies in CS [19]. STE enhances echocardiographic sensitivity because research indicates that GLS measurements identify CS cases not detected by standard echocardiographic parameters [20]. Strain measurements are increasingly used in sarcoidosis screening because reduced GLS and specific regional strain patterns prompt further diagnostic testing.

Diastology is another echocardiographic technique used by cardiologists to detect CS, as it leads to diastolic dysfunction through granuloma formation and fibrosis, manifesting as elevated E/e’ ratios and impaired relaxation patterns [21]. The identified patterns occur frequently in other diseases, rendering them of limited diagnostic value. Echocardiographic measures such as tricuspid annular plane systolic excursion (TAPSE) and RV fractional area change assess RV systolic function and may be abnormal in patients with CS; however, these findings are nonspecific and may reflect secondary effects such as pulmonary hypertension from pulmonary sarcoidosis, ventricular interdependence or advanced myocardial involvement rather than direct granulomatous infiltration of the RV [22]. Echo‐based electromechanical mapping methods enable researchers to assess interventricular mechanical delays and other indicators of dyssynchrony resulting from conduction disturbances caused by CS.

Most echocardiographic findings, including reduced EF and septal thinning, are nonspecific because they can result from other cardiomyopathies. The diagnosis of CS can be made only by combining a known diagnosis of sarcoidosis with the exclusion of other possible causes based on these findings. Poor image quality in some patients, due to obesity or lung disease, reduces the visibility of the regional conclusions. Strain imaging shows promise; however, its effectiveness depends on image quality, and standardisation for defining specific CS patterns remains limited among vendors. The myocardial wall remains invisible to echocardiography, but the technique can detect granulomas and fibrosis by observing their secondary effects. A small basal aneurysm may be missed on an echocardiography because it requires the operator’s specific attention and expertise. Echocardiography does not provide evidence of scar formation or inflammation within the myocardial wall; therefore, CMR or PET scans are required to establish active CS and determine its extent. No single imaging test is definitive for CS. Histologic confirmation is the reference standard, but unguided endomyocardial biopsy has limited sensitivity because myocardial involvement is focal and patchy. TTE remains a key first‐line tool for functional assessment and follow‐up, while CMR and FDG PET are central to demonstrating myocardial involvement and disease activity [23, 24]. Lastly, TTE provides an ideal tool for ongoing evaluations because it enables simple, repeated EF assessments to monitor the effects of cardiotoxic therapies on function and to identify progression that requires advanced imaging.

### 2.1. CMR

The combination of CMR with LGE has established itself as the standard method for assessing CS because it delivers precise scar imaging alongside radiation‐free tissue characterisation [8, 25]. The main advantage of CMR is its noninvasive nature and lack of radiation exposure, which enable multiple assessments as needed. A single CMR assessment provides detailed results on fibrosis and oedema tissue characterisation, ventricular performance and cardiac structure visualisation. CS most commonly manifests as nonischaemic patterns on LGE, typically involving scattered midmyocardial and subepicardial enhancement affecting the basal septum, inferolateral walls and papillary muscles. However, ischaemic‐appearing enhancement patterns have also been reported. In a landmark study by Patel et al., approximately 14% of the patients with CS showed LGE patterns consistent with coronary artery disease, underscoring that LGE alone is not specific and should be interpreted in the context of clinical findings and complementary imaging [26]. The observed imaging findings represent fibrotic or granulomatous replacement of myocardial tissue. Imaging of oedema using T2‐weighted sequences and newer parametric mapping techniques, such as T1/T2 mapping, can detect active inflammation or granulomas as areas of elevated signal or prolonged relaxation times [27]. LGE in CS typically involves the septum and the lateral/free wall and sometimes extends to the right ventricle. The LGE patterns in CS do not conform to the typical distribution of infarction scars, as they avoid the subendocardial layer and do not align with the territory patterns of the coronary arteries. Assessing biventricular dimensions alongside cardiac function and wall motion patterns, and detecting ventricular aneurysms and wall thinning on CMR, helps support CS diagnosis when patients present with appropriate clinical signs. The additional techniques of T1 mapping and extracellular volume (ECV) quantification enable detection of diffuse interstitial fibrosis or inflammation beyond focal LGE, potentially improving sensitivity. T1 mapping enables researchers to detect CS in patients without LGE on imaging, according to a previous study. CMR imaging techniques help medical professionals guide biopsies by identifying specific scar locations that maximise success rates.

Diagnostic performance: CMR has shown excellent sensitivity for CS. Multiple studies have analysed CMR performance in detecting CS through meta‐analysis, yielding sensitivities of 90%–95% and specificities of 80%–90% [8]. The exceptional detection performance of CMR for myocardial involvement matches autopsy detection rates and surpasses conventional screening methods. CMR enables healthcare providers to identify heart problems in patients who do not exhibit signs of heart disease or have received initial standard testing results. The study by Kouranos et al. of 321 sarcoidosis patients found that CMR was the most valuable test for diagnosing and predicting CS outcomes [16]. The study revealed that CMR identified 59 cases of CS but would have remained undetected by standard screening procedures, including 15 asymptomatic patients with normal electrocardiogram (ECG) and echocardiogram results. The sensitivity of echocardiography reached only 27% in that specific study. The results demonstrate that CMR successfully identifies previously unrecognised cases of CS. The high negative predictive value of normal CMR results makes it a recommended screening tool for patients with systemic sarcoidosis who have risk factors [28]. The study conducted by Murtagh et al. found that out of 205 sarcoidosis patients with normal EF, 20% showed LGE on CMR, and LGE‐positive patients faced more than a 20‐fold increased annual risk of death or sustained ventricular tachycardia (VT) compared with LGE‐negative patients, who had a 0.2% per year risk [29]. The annual risk of death or ventricular arrhythmia increased by approximately 8%, with each 1% increase in myocardial LGE burden. Research conducted on 155 patients across multiple centres demonstrated that patients with CMR scar faced a substantially higher risk of death, along with appropriate intracardiac defibrillator (ICD) shocks and aborted sudden death (hazard ratio ∼31 on multivariate analysis) when compared with patients without scar [30]. A recent study involving 694 patients demonstrated that the presence of LGE in the heart leads to cardiovascular death risks that are 10 times higher and ventricular arrhythmia risks that are 20 times higher when compared with LGE‐negative patients [28]. Event rates among patients without LGE were extremely low, supporting the conclusion that a negative CMR result indicates a favourable prognosis. The practical value of CMR findings, particularly the extent and location of LGE, has become a standard consideration in ICD implantation decisions for patients with CS, regardless of the EF status [31, 32]. We have aligned our description with the 2017 AHA/ACC/HRS Guideline for Management of Patients With Ventricular Arrhythmias and the Prevention of Sudden Cardiac Death, which includes Class IIa recommendations for ICD implantation in selected patients with CS, such as those with EF greater than 35% and myocardial scar, syncope suggestive of arrhythmic origin, inducible ventricular arrhythmia on electrophysiology study or an indication for permanent pacing. These recommendations are based on observational evidence and expert consensus rather than prospective randomized trials [33].

The technique is subject to certain restrictions that affect its practical use. The traditional rule against magnetic resonance (MRI) scans for patients with cardiac devices, such as pacemakers and defibrillators, no longer applies, as most modern devices are MRI‐compatible and specific protocols exist for patients with these devices. Device‐related artefacts degrade image quality, particularly when the device is located in the anterior chest. CMR may be limited in patients with advanced kidney disease due to concerns regarding gadolinium‐based contrast administration; however, contemporary guidelines permit the use of newer, lower‐risk gadolinium agents in patients with chronic kidney disease and end‐stage renal disease when clinically indicated. The LGE imaging technique detects fibrosis and possibly active granulomatous inflammation but cannot distinguish active inflammation from chronic scarring. The method demonstrates excellence in identifying the location and presence of cardiac involvement; however, it fails to distinguish between active and inactive conditions. CMR provides superior detection of cardiac involvement and its location but fails to differentiate between active and inactive conditions. Clinical and imaging findings should be consistent to determine the active disease status, as indicated by the presence of new LGE and LGE with oedema. The CMR imaging procedure requires patients to remain still and breathe normally during ECG gating; however, patients with arrhythmias often exhibit suboptimal image quality. Additionally, patients who cannot lie flat or stay still may experience difficulties during the exam. Figures 1 and 2 illustrate CMR findings in CS.

FIGURE 1Cardiac magnetic resonance of a patient in his 40s presenting with left ventricular impairment and frequent ventricular ectopy. (a) Late gadolinium enhancement in the basal anteroseptum, extending to the basal anterior wall, mid inferoseptum and mid inferior wall (blue arrows). (b) T2 mapping with increased signal in the midinferolateral wall.

**Figure:**
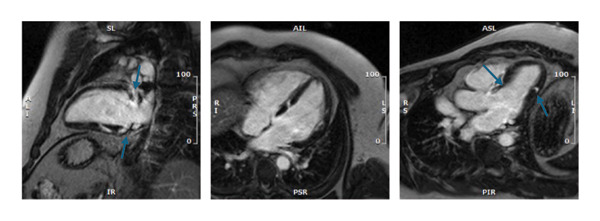
(a)

**Figure:**
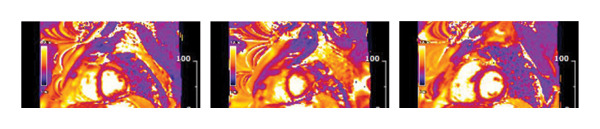
(b)

FIGURE 2Cardiac magnetic resonance for a 25‐year‐old male who presented with haemodynamically untolerated ventricular tachycardia requiring two direct current cardioversions. (a) Late gadolinium enhancement in the mid and basal septum. There was also epicardial fibrosis in the midinferior and the RV side of the midseptum and midlateral wall (blue arrows). (b) Increased T2 signals in the midlateral and midanteroseptal walls.

**Figure:**
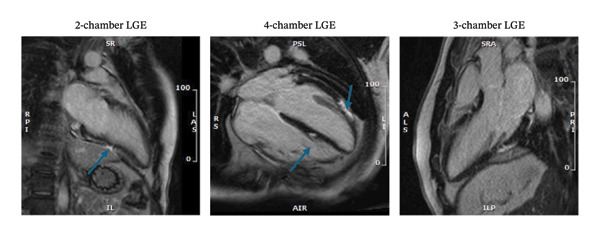
(a)

**Figure:**
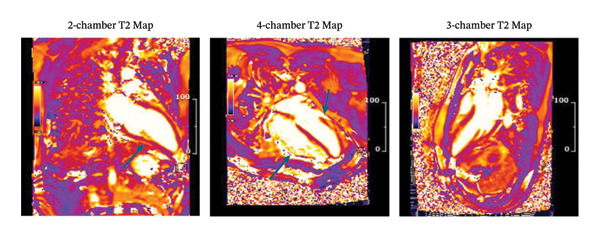
(b)

### 2.2. PET

The application of FDG‐PET/CT exploits elevated glucose metabolism within sarcoid granulomas, which predominantly consists of activated macrophages [25]. Patients receive specialised preparation involving a high‐fat diet with minimal carbohydrates, combined with fasting, to reduce baseline cardiomyocyte glucose uptake, thereby allowing pathologic activity to become evident. Following FDG injection, PET imaging distinguishes regions of active inflammation as focal hotspots of FDG uptake, consistent with the patchy distribution of granulomatous lesions. The PET examination requires the addition of low‐dose CT, which performs attenuation correction and anatomical localisation. Many diagnostic protocols include myocardial perfusion imaging with PET tracers such as Rubidium‐82 or N‐13 ammonia, or SPECT tracers such as 201Tl or 99mTc, which are often performed during the same procedure [7]. The procedure helps healthcare professionals detect perfusion defects. The typical CS pattern presents as a perfusion‐metabolism mismatch, characterised by increased FDG uptake in areas exhibiting reduced perfusion, microvascular impairment and scar. The imaging capability of PET/CT extends to whole‐chest and whole‐body examinations, enabling simultaneous detection of extracardiac sarcoidosis [34, 35].

Multiple studies have shown that FDG‐PET demonstrates excellent detection of active sarcoid inflammation in the heart. A 10‐year review of the medical literature indicates that PET diagnostic accuracy reaches approximately 80% for identifying CS. A comprehensive analysis of 17 studies, including 891 patients, demonstrated that PET had 84% sensitivity and 83% specificity for detecting CS [36]. Furthermore, a meta‐analysis of 164 patients across seven studies reported FDG‐PET sensitivity of 89% and specificity of 78% when evaluated against Japanese pathological criteria [37]. The high sensitivity of PET indicates that active CS becomes detectable with appropriate imaging; false negatives primarily result from poor patient preparation and inactive disease states. PET specificity varies across cases because various cardiac FDG uptake patterns do not necessarily indicate disease. False‐positive results on FDG‐PET can arise from physiological and inflammatory uptake from multiple etiologies, such as atypical myocarditis and hibernating myocardium in ischaemic conditions. FDG uptake in a nondiabetic patient who underwent appropriate dietary preparation strongly indicates CS when the clinical context is relevant. The HRS criteria now utilise “patchy uptake on FDG‐PET” as one of the major criteria for probable CS when systemic sarcoidosis is present [5]. PET/CT is particularly useful for diagnosing solitary CS when extracardiac biopsy is not feasible, as PET imaging findings of inflammation, combined with CMR data and clinical evidence, can establish the diagnosis under Japanese guidelines [38].

The diagnostic confidence level rises when perfusion and metabolic imaging procedures are combined. A specific sign of active CS is the presence of areas that exhibit both reduced perfusion and increased FDG uptake. The appearance of uniform perfusion defects and FDG uptake in a region indicates active inflammation of scarred myocardium. However, matched defects without FDG uptake suggest chronic scar, and isolated FDG uptake with normal perfusion indicates early inflammation without fibrosis [39]. Multiple healthcare facilities offer PET perfusion scanning as an integrated procedure.

RV FDG uptake occurs infrequently in other medical conditions, yet its presence in CS disease indicates extensive disease progression [40]. The ability of FDG‐PET to monitor inflammation is evident from successive imaging results in a patient with CS before and after treatment. FDG‐PET findings also carry prognostic information. Several studies have shown that the presence and extent of cardiac FDG uptake are associated with worse outcomes. In a cohort of 118 suspected CS patients, those with any abnormal cardiac FDG uptake plus a perfusion defect had a fourfold higher annual risk of death or VT than those without these findings. Notably, the patients with the mismatch pattern (perfusion defect with FDG uptake) are at a high risk for arrhythmias. Moreover, focal RV uptake has been associated with a fivefold increase in risk of death or sustained VT and is believed to indicate advanced disease [41]. Quantitative analysis of FDG‐PET may improve risk stratification in CS. Higher standardized uptake values (SUV) and a greater number of FDG‐avid segments have been associated with worse outcomes, including major adverse cardiac events and all‐cause mortality, supporting the incremental prognostic value of quantitative PET metrics. Quantitative PET also has utility in serial imaging to monitor treatment response and disease activity over time. Serial changes in SUV and the extent of FDG uptake have been shown to reflect therapeutic response, with decreases in quantitative uptake correlating with clinical improvement and reduced inflammatory burden on follow‐up scans [42].

Another important role of PET is therapy monitoring. Since FDG uptake diminishes with successful immunosuppressive treatment, as granulomatous inflammation resolves, serial PET scans can be used to assess treatment response. Studies have shown that the reduction or normalisation of cardiac FDG uptake is associated with better outcomes and has been used to guide steroid tapering [43]. The current expert statements recommend that follow‐up FDG‐PET scans be used to assess disease activity in patients with CS undergoing therapy [44]. PET can thus be employed to optimise immunosuppression. For instance, treatment should be continued until cardiac FDG uptake clears, preventing discontinuation and subsequent relapse. This is an area of ongoing research, including studies to determine whether PET‐guided therapy yields better clinical outcomes.

Despite its strengths, FDG‐PET/CT has important limitations. First, the patient must be well prepared to suppress physiological myocardial glucose uptake. If preparation fails (which can occur in up to 20–25% of the patients), the resulting diffuse uptake can mask pathological foci or lead to false positives [44]. Patients are required to follow a strict high‐fat, low‐carbohydrate diet for 12–24 h, often with an overnight fast of 12–18 h. Even with proper preparation, normal variants (such as basal uptake or RV uptake, especially in younger patients or those with insufficient fasting) can complicate interpretation. Second, PET shows inflammation but not fibrosis; therefore, it cannot detect myocardial scar or damage. Some cases of “burnt‐out” CS (fibrosis without active inflammation) will be PET‐negative but CMR‐positive. Thus, a negative PET does not rule out prior CS if fibrosis persists (and this is why PET and CMR are valuable). Another limitation is specificity: conditions such as acute myocarditis from other causes, cardiac infections or even physiologic uptake in hypertrophied myocardium can mimic CS uptake patterns. Careful interpretation and correlation with CMR or clinical data are necessary to prevent misdiagnosis [42, 44]. Interpretation of cardiac PET can also be challenging, as distinguishing between “focal” and “diffuse” uptake (which may represent incomplete suppression) and true focal uptake is subjective. However, standardised criteria (e.g., SNMMI/ASNC) help define an abnormal pattern (focal or focal‐on‐diffuse considered positive in context). PET imaging is relatively costly and less prevalent than echocardiography; it also entails radiation exposure from the radiotracer and from CT scanning. The typical dose of FDG cardiac PET/CT is 7.15 mSv, which is a consideration, especially if repeated scans are planned; however, it remains within diagnostic ranges and is justified given the severity of CS. Finally, although PET spatial resolution is good, it is inferior to that of CMR. Very small granulomas (< 5 mm) may fall below the detection threshold, and PET cannot map the exact myocardial layer of uptake as precisely as CMR.

Before PET became widely used, nuclear imaging of CS relied on SPECT techniques, such as perfusion scintigraphy and gallium scintigraphy. SPECT works by detecting gamma‐emitting tracers through rotating cameras to produce 3D images. Myocardial perfusion SPECT reveals regional perfusion defects when performed at rest or during stress in CS [45]. Granulomatous infiltration, along with microvascular disruption, results in patchy areas of recondensation in the CS. According to the literature, perfusion defects associated with sarcoidosis are typically stable (unlike ischaemia), exhibit reverse distribution (worse at rest than during stress uptake) and are visualised with 201Tl imaging [46]. When sarcoid lesions replace myocardial tissue, they produce areas of reduced radiotracer uptake on perfusion imaging, reflecting regional myocardial damage or scarring. Perfusion defects commonly occur in regions outside the coronary artery territories, such as the basal septum and lateral wall, making CS more likely than coronary artery disease. When PET perfusion tracers are unavailable, patients can use SPECT perfusion followed by PET‐FDG imaging in a sequential order: SPECT for perfusion and then PET for metabolism to detect mismatches.

SPECT imaging uses Gallium citrate‐67 (^67^Ga) as its radiopharmaceutical. Sarcoid inflammation detection began with ^67^Ga as the first radionuclide because this element accumulates in inflammatory areas through binding to transferrin and lactoferrin in inflammatory cells [47]. In previous decades, planar or SPECT imaging of cardiac gallium uptake was used to demonstrate active CS. Gallium SPECT has lost popularity as a diagnostic tool over the past decade. The sensitivity of gallium SPECT is inferior to that of FDG‐PET, and image resolution is poor because gallium imaging produces coarse images during long acquisition times, which require high radiation doses. Due to its superior performance, most medical centres have chosen to use FDG‐PET instead of gallium for imaging of inflammation [48]. Beyond FDG‐PET and historical gallium‐67 SPECT imaging, newer gallium‐68‐based PET tracers are emerging as promising tools for CS evaluation. Gallium‐68 DOTATATE targets somatostatin receptor subtype‐2 expressed on activated macrophages within granulomas and may offer improved specificity compared with FDG by avoiding physiological myocardial uptake. Early studies suggest that 68Ga‐DOTATATE PET can identify active CS without requiring complex dietary preparation and may be particularly useful in cases with equivocal FDG‐PET. Gallium‐68 citrate PET has also been explored as an inflammation‐targeting tracer, although clinical experience remains limited. More recently, gallium‐68 labelled fibroblast activation protein inhibitor (FAPI) PET has been proposed as a novel approach to visualise myocardial fibroblast activation and fibrotic remodelling, potentially complementing inflammation‐focused imaging. While these tracers remain investigational and are not yet incorporated into routine clinical guidelines, they represent an important area of ongoing research. They may play a future role in improving diagnostic specificity and phenotyping of CS.

SPECT technology continues to serve useful functions in specific clinical situations. The evaluation process can benefit from SPECT when PET is unavailable. The presence of resting perfusion defects on SPECT in patients with sarcoidosis without obstructive coronary artery disease suggests cardiac involvement. SPECT and PET can be performed on hybrid SPECT/CT systems, which enable simultaneous acquisition of SPECT perfusion and PET‐FDG scans in a single session. Other aspects can be evaluated using SPECT, which allows imaging of 123I‐metaiodobenzylguanidine (MIBG) in cardiac sympathetic innervation and its application in cardiac sympathetic studies. Research findings indicate that denervation regions in CS can be identified by reduced MIBG uptake; however, MIBG testing is not the standard for diagnosing CS [49]. The SPECT imaging agent ^111In‐pentetreotide serves as an octreotide scintigraphy probe to detect active somatostatin receptors in macrophages [50]. The detection of sarcoid inflammation was possible using ^111In‐octreotide as a tracer, even before PET technology became available. However, the results were not outstanding [51].

SPECT is a contemporary diagnostic option when PET or CMR imaging is unavailable. The imaging procedure for an ICD patient with poorly controlled diabetes, without CMR, typically involves ^201Tl or ^99mTc perfusion SPECT and a ^67^Ga scan. Results from perfusion defects and gallium uptake will support the diagnosis of CS; however, negative results will require additional evaluation in the setting of a strong clinical suspicion. Future developments in PET tracers targeting somatostatin receptors may lead to SPECT agents utilising ^111In or ^99mTc isotopes for sarcoid detection; however, PET remains the dominant direction in medical imaging.

### 2.3. CT

Cardiac CT has a complementary role in the assessment of CS, particularly in patients with contraindications to CMR or with cardiac implantable electronic devices. Delayed enhancement cardiac CT, also referred to as late iodine enhancement CT, enables visualisation of myocardial scar using iodinated contrast. Although protocols are not fully standardised, delayed imaging is typically performed 5–10 min after contrast administration, often following an initial coronary CT angiography acquisition. ECG‐gated acquisition with iterative reconstruction is commonly used to optimise image quality, and, when available, dual‐energy or spectral CT techniques may improve scar detection by enhancing contrast differentiation. Emerging evidence suggests that delayed enhancement CT can identify myocardial involvement in CS and may demonstrate scar patterns comparable to CMR in selected patients, supporting its use as an alternative imaging modality when CMR is not feasible [52, 53]. Because myocardial scar, wall motion abnormalities and perfusion defects may also result from ischemic heart disease, the exclusion of obstructive coronary artery disease is essential when interpreting advanced imaging findings in patients with suspected CS. This is typically achieved using invasive coronary angiography or coronary CT angiography, depending on clinical context.

### 2.4. Hybrid and Multimodality Imaging

CS often requires a multimodal approach for optimal diagnosis and management, as no single test provides a comprehensive picture of scar formation, inflammation and functional impact. Hybrid imaging refers to the simultaneous acquisition of data from two modalities or to the integrative interpretation of multiple imaging datasets. The most pertinent hybrid approach in CS is PET combined with CMR, which merges metabolic and structural imaging. This can be achieved by software fusion of separately acquired CMR and PET images, or by using integrated PET/MR scanners now available in specialised centres [54]. Simultaneous PET/MR imaging can accurately coregister inflammation and fibrosis in a single session. A recent landmark study by Greulich et al. prospectively evaluated 43 patients with suspected CS on a 3T PET/MR scanner [54]. After appropriate preparation, each patient underwent comprehensive CMR (cine, LGE, and T1‐ and T2‐mapping for tissue characterisation) concurrently with FDG‐PET. The researchers defined “active CS” as cases with both PET and CMR evidence of disease (PET+/CMR+, indicating active inflammation in areas of scar/oedema) and “chronic CS” as CMR‐positive scar without PET uptake (PET,/CMR+). At the same time, PET negativity and CMR negativity had no CS. Using this approach, they found that 36% of the patients had active CS and 14% had chronic (inactive) CS, whereas 50% had no CS. Notably, CMR mapping techniques increased the detection yield in 22% of LGE‐negative CS patients; abnormal T1 mapping still identified myocardial involvement. This underscores that combining advanced CMR with PET can catch cases that a single modality might miss. Importantly, the hybrid imaging allowed differentiation of disease activity: those with PET uptake were considered candidates for immunosuppressive therapy, whereas those with only scar (no PET uptake) might represent burned‐out disease, less likely to benefit from anti‐inflammatory treatment. The authors concluded that integrated PET/MR “provides additional value for identifying active disease” and could improve diagnosis and management of CS.

In practical terms, hybrid PET/MR offers an all‐in‐one test: patients undergo a single scan, receiving both an LGE and an inflammation assessment. This is convenient and ensures optimal timing (the LGE and PET correspond to the same physiologic state). It can also reduce overall radiation exposure by eliminating the need for CT attenuation correction since MRI sequences can be used to generate attenuation maps.

Even without an integrated scanner, combining information from separate CMR and PET scans is standard in CS evaluation. Clinicians often obtain a CMR for scar assessment, followed by PET to look for inflammation (or vice versa). The complementary nature is that a patient with positive LGE but negative FDG uptake might be diagnosed with probable CS (if systemic sarcoidosis is present) but might not require high‐dose immunosuppression at that time (because there is no active inflammation). On the other hand, a patient with significant FDG uptake but minimal LGE may be in an early inflammatory stage, potentially reversible with treatment, and the goal would be to prevent progression to fibrosis. Integrative interpretation schemes have been proposed. For instance, one algorithm assigns each patient a probability of CS (none, possible, probable, or highly probable) based on combined imaging findings [20]. In one series of 107 patients, adding FDG‐PET results to CMR findings reclassified 45% of patients into a different likelihood category. Some patients were upgraded because PET detected inflammation where CMR was negative, while others were downgraded because PET was clean despite CMR LGE [55]. This demonstrates that discordant findings are common and each modality contributes unique information. Hybrid imaging can also refer to PET/CT, an integrated modality routinely used in CS to map perfusion (via CT or SPECT) alongside FDG uptake. Some centres perform same‐day PET/CT and CMR (back‐to‐back) and utilise software to fuse the images.

Isolated CS, defined as cardiac involvement without histologic confirmation of extracardiac sarcoidosis, remains a diagnostically challenging condition. The 2014 HRS criteria require myocardial tissue confirmation for a definite diagnosis and do not provide a standalone imaging‐based pathway for isolated CS [5]. In contrast, the Japanese Circulation Society criteria permit a clinical diagnosis of isolated CS based on combinations of major criteria that incorporate CMR and FDG‐PET findings, reflecting real‐world reliance on advanced imaging when extracardiac biopsy is negative or not feasible [6]. In clinical practice, CMR and FDG‐PET are used as complementary tests in suspected isolated CS, where PET positivity may reflect early inflammatory disease, and CMR may demonstrate scar patterns that support myocardial involvement. Multimodality imaging recommendations and flowcharts from joint society statements emphasise this complementary approach, particularly when biopsy yield is low.

**FIGURE 3:**
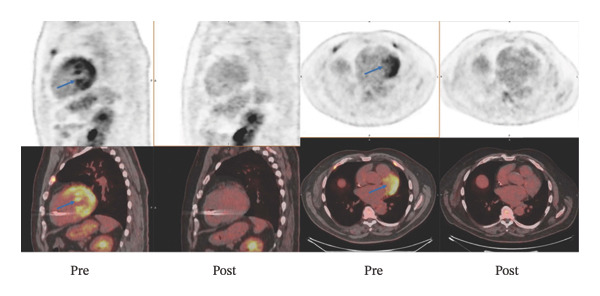
Representative sagittal and axial PET and PET/CT fusion images in a patient with cardiac sarcoidosis. The top row shows baseline PET images with increased FDG uptake in the left ventricular myocardium (blue arrows), indicating active inflammation. The bottom row displays corresponding fused PET/CT images. Posttreatment scans (second and fourth columns) demonstrate a significant reduction in myocardial FDG uptake, consistent with the metabolic response to immunosuppressive therapy.

Additionally, electrophysiological mapping data can be integrated with imaging (e.g., to guide VT ablation in CS, scar maps from CMR or PET can be overlaid onto electroanatomic maps). While beyond the scope of this review, it is worth noting that multimodality integration is central to the management of arrhythmias in CS. Figure 3 demonstrates PET scans before and after treatment.

Somatostatin receptor PET imaging with 68Ga‐DOTATATE can be performed on standard PET/CT systems and does not require hybrid PET/MR imaging. While PET/MR may offer advantages by enabling simultaneous assessment of inflammation and myocardial scar, 68Ga‐DOTATATE PET/CT alone can identify inflammatory activity and has been explored as an alternative to FDG‐PET in selected cases [56, 57]. Future hybrid imaging may involve PET/MR with such tracers to map scar and, concurrently, provide a more specific signal for inflammation.

The synergy of modalities improves patient care. For example, a patient with high‐grade atrioventricular block and extracardiac sarcoidosis might undergo MRI that shows LGE in the septum and PET that shows FDG uptake in the same area. This confirmation of active CS would lead to steroid therapy and possibly a pacemaker/ICD. If either test was negative, the diagnosis or treatment plan might change (if the PET scan was negative, observation or a different diagnosis might be considered). Hybrid imaging thus refines diagnosis, prevents over‐ or undertreatment and can guide interventions (like device implantation decisions).

**FIGURE 4:**
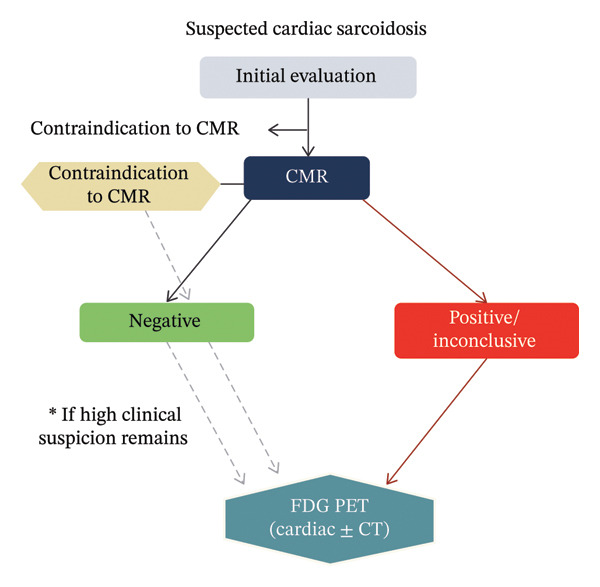
Multimodality imaging algorithm for suspected cardiac sarcoidosis. Patients with suspected CS undergo initial evaluation followed by CMR when available. A positive or inconclusive CMR prompts FDG PET to assess active myocardial inflammation. If CMR is negative but clinical suspicion remains high, FDG PET is recommended to detect early inflammatory disease that may precede fibrosis. In patients with contraindications to CMR, FDG PET with or without CT serves as an alternative diagnostic pathway. This algorithm highlights the complementary roles of CMR for scar assessment and FDG PET for inflammatory activity.

Occasionally, advanced imaging modalities may yield discordant results, such as a positive FDG‐PET scan with evidence of active inflammation and a negative CMR scan without LGE. This pattern may arise because PET identifies metabolic activity associated with early granulomatous inflammation before irreversible fibrosis has developed, whereas CMR LGE primarily detects scar and fibrotic change. Thus, a positive PET with negative CMR may suggest early or predominantly inflammatory disease without significant fibrosis, a phase in which anti‐inflammatory treatment might be most beneficial. Conversely, a positive CMR with negative PET can indicate a scar that is no longer metabolically active and may represent quiescent disease or healed granulomas. These discordant patterns emphasise the complementary roles of PET and CMR and underscore the importance of interpreting imaging in the context of the clinical presentation, ECG and other biomarkers [41, 58]. Table 1 compares the previously mentioned imaging modalities for CS. A practical imaging algorithm for suspected CS is as follows. TTE should be performed at baseline in all patients to assess function. CMR should be the first advanced imaging modality available, given its high sensitivity for myocardial scar detection and its utility for prognostic stratification. If CMR demonstrates LGE, FDG‐PET should be performed to assess active inflammation and guide immunosuppressive therapy. If CMR is negative but clinical suspicion remains high, FDG PET should be considered to detect early inflammatory disease. PET MR may be used in specialised centres when simultaneous assessment of scar and inflammation is required, particularly in equivocal cases. In patients with contraindications to CMR, FDG‐PET with or without CT provides an alternative diagnostic pathway [55, 59] (Figure 4).

**TABLE 1:** Summary of imaging modalities in cardiac sarcoidosis: diagnostic performance, strengths, limitations and prognostic value.

Imaging modality	Key findings/role	Sensitivity	Specificity	Strengths	Limitations	Prognostic value
Echocardiography (TTE, STE)	Structural/functional assessment; initial screening	Low (∼25%)	Moderate	Easily accessible, real time, portable	Nonspecific; operator dependent; misses small lesions	GLS predicts early myocardial dysfunction
Cardiac MRI (CMR/LGE)	Scar detection; structural assessment	High (90%–95%)	High (80%–90%)	No radiation; detailed tissue characterisation	Limited by arrhythmia, kidney dysfunction; no inflammation differentiation	Strongly predicts arrhythmias and mortality
FDG PET/CT	Active inflammation; treatment response	High (∼85%)	Moderate (∼80%)	Detects active inflammation, monitors therapy	Dietary preparation required; radiation; false positives possible	Predicts risk of arrhythmias and mortality
Hybrid Imaging (PET/MR)	Combined scar and inflammation assessment	High	High	Comprehensive; one‐session scan; improves diagnosis	Costly; limited availability	Clearly differentiates active from inactive disease
CT (Delayed‐enhanced)	Scar assessment when MRI contraindicated	Moderate	Moderate	Suitable for device patients; excludes CAD	Radiation exposure; lower tissue contrast	Limited direct prognostic evidence

*Note:* TTE: transthoracic echocardiography; FDG: fluorodeoxyglucose.

Abbreviations: CAD, coronary artery disease; CMR, cardiac magnetic resonance; CT, computed tomography; GLS, global longitudinal strain; LGE, late gadolinium enhancement; PET, positron emission tomography; SPECT, single‐photon emission computed tomography; STE, speckle‐tracking echocardiography.

### 2.5. Artificial Intelligence (AI) in Cardiac Sarcoidosis Imaging

AI and machine learning (ML) are emerging as transformative tools in cardiology [60–63], more specifically, cardiac imaging and CS assessment [64]. AI applications in CS imaging have focused on enhancing diagnostic accuracy, standardising image interpretation and integrating multimodal data for improved risk stratification.

Recent studies have demonstrated that AI algorithms can assist in the automated detection of myocardial scars on CMR, thereby improving reproducibility and potentially identifying subtle LGE patterns that human observers may miss. Similarly, in PET, ML models trained on standardised uptake values and the spatial distribution of FDG uptake have shown promise in differentiating active sarcoid inflammation from physiologic or artefact‐related uptake [65].

Multimodal fusion algorithms are also being developed to integrate CMR, PET and clinical data to predict adverse outcomes such as ventricular arrhythmias or progression to heart failure. As datasets grow and become more standardised, AI is expected to play an increasing role in CS imaging, from automating lesion segmentation to enabling real‐time interpretation during hybrid PET/MR scans. However, current limitations include the scarcity of large, labelled datasets specific to CS and the need for external validation across diverse populations.

### 2.6. Limitations of the Current Literature

Recognising all these factors is essential. Most cohort studies use single‐centre cohorts and are subject to selection bias, as patients may have CS. Reference standards differ, whether based on clinical criteria or expert consensus, making it difficult to compare the modality’s performance directly. There is overlap in what the modalities can detect (scar vs. inflammation), and this will depend on the reference used (e.g., some PET “false positives” may reflect early CS that the reference standard missed). Studies on the prognostic value will be more useful if the gold standards are applied prospectively (i.e., based on advanced imaging or long‐term outcomes). Most prognostic studies have a moderate sample size; hence, larger registries or pooled data analyses are needed to achieve more precise risk stratification, especially to determine whether treating subclinical CS (imaging‐detected only) improves outcomes, an issue that has not been addressed yet.

From a clinical perspective, synthesising the findings is crucial. A practical imaging strategy for suspected CS is to perform CMR first, when available. If CMR demonstrates LGE, FDG‐PET should follow to determine active inflammation. When CMR is negative but clinical suspicion remains high (for example, in patients with extracardiac sarcoidosis or concerning arrhythmias), FDG‐PET should still be considered because PET can detect inflammation before structural changes become apparent on CMR. This approach is supported by expert imaging consensus and diagnostic flowcharts.

Finally, patient‐centred considerations should not be ignored. This can burden patients, including the need for multiple scans, fasting prior to PET and contrast medium administration. Educating patients about why each test is necessary and how it complements others will enhance compliance. Additionally, the psychosocial impact of this should not be overlooked; patients may be alarmed to see FDG uptake on a scan, often confusing it with cancer imaging; thus, the results need to be explained to them in a manner they can understand.

## 3. Conclusion

Medical imaging in CS has evolved from a secondary tool to the primary diagnostic and management tool. The two tests, CMR and PET/CT, have established themselves as the standard of care for detecting myocardial scar and active inflammation and have improved CS detection compared with previous methods. CMR technology provides excellent information on the presence and extent of cardiac fibrosis, which helps inform prognostic predictions and plan ICD therapy. The FDG‐PET/CT scan reveals active granulomatous inflammation and helps assess the effectiveness of immunosuppressive treatment. A comprehensive multimodality toolkit includes echocardiography for functional assessment and screening, SPECT for perfusion abnormalities or when PET is unavailable and cardiac CT for patients with contraindicated CMR. Integrating these techniques through hybrid imaging improves diagnostic accuracy and differentiates active disease from postinflammatory scarring.

Studies and meta‐analyses from the last decade have established the importance of these modalities: LGE on MRI and focal FDG uptake on PET are now major diagnostic criteria for CS and strong predictors of future adverse events. Modern CS management requires advanced imaging, as clinicians use imaging results to determine when to initiate or intensify immunosuppressive therapy, when to implant devices for arrhythmia prevention and how to monitor disease progression. The interdisciplinary care of CS patients, often treated by cardiologists, electrophysiologists, radiologists and sarcoidosis specialists, depends heavily on imaging results.

Cardiac imaging has revolutionised the diagnosis of CS, shifting it from an exclusion‐based to an imaging‐based approach that can be made without invasive procedures and tracked over time. The combination of imaging modalities enables physicians to more comprehensively assess the extent and activity of disease in each patient. The treatment approach can be individualised by aggressively treating inflammation to prevent progression of fibrosis in patients at high risk of arrhythmias, while avoiding overtreatment in patients with inactive disease. New tracers, mapping techniques and integrated imaging platforms are expected to enhance our diagnostic precision and prognostic capabilities. The information obtained from imaging studies enables the delivery of appropriate and timely treatment to patients with this treatable but potentially lethal condition. Multimodality imaging in CS underscores the significant impact of advanced cardiovascular imaging on patient care and guides future developments in this field.

## Author Contributions

Conceptualisation, Ibrahim Antoun and Sanjay S. Bhandari; methodology, Ibrahim Antoun and Sanjay S. Bhandari; writing–original draft preparation, Ibrahim Antoun; writing–review and editing, Ibrahim Antoun, Sanjay S. Bhandari, Mustafa Zakkar, and G. Andre Ng.

## Funding

No funding was received for this research.

## Ethics Statement

The authors have nothing to report.

## Conflicts of Interest

The authors declare no conflicts of interest.

## Data Availability

Data sharing is not applicable to this article as no new data were created or analysed in this study.
